# Covid-19-related stressors, mental disorders, depressive and anxiety symptoms: a cross-sectional, nationally-representative, face-to-face survey in Serbia

**DOI:** 10.1017/S2045796022000117

**Published:** 2022-05-24

**Authors:** N. P. Marić, L. J. B. Lazarević, S. Priebe, L. J. Mihić, M. Pejović-Milovančević, Z. Terzić-Šupić, O. Tošković, O. Vuković, J. Todorović, G. Knežević

**Affiliations:** 1 University of Belgrade, Faculty of Medicine, Belgrade, Serbia; 2 Institute of Mental Health, Belgrade, Belgrade, Serbia; 3 University of Belgrade, Faculty of Philosophy, Belgrade, Serbia; 4 Unit for Social and Community Psychiatry (WHO Collaborating Centre for Mental Health Service Development), Queen Mary University of London, London, UK; 5 University of Novi Sad, Faculty of Philosophy, Novi Sad, Serbia

**Keywords:** Common mental disorders, stresful life events, population survey, mental health, risk factors, NCT 04896983

## Abstract

**Aim:**

The Covid-19 pandemic may be associated with an increase in mental disorders and mental distress. However, there are no representative studies testing the impact of stressors directly related to Covid-19. We aimed to determine whether Covid-19-related stressors were associated with mental disorders, depressive and anxiety symptoms in the second year of the pandemic.

**Method:**

This cross-sectional observational epidemiological survey was conducted from June to October 2021. We interviewed a representative sample of the adult population in Serbia (18–65 years) in the second year of the pandemic, at a time when large parts of the population had been affected by the pandemic in different ways. A multistage probabilistic household sampling of the adult population in 60 municipalities was used. Mental disorders were assessed by in-person interviews using the Mini International Neuropsychiatric Interview. Depressive and anxiety symptoms were measured by PHQ-9 and GAD-7 scales. Covid-19-related stressors (Sars-CoV-2 infection, the infection of a close relative, self-isolation and lack of protective equipment at work), as well as other stressors during the pandemic (not directly related to the risk of the infection), were measured. The associations with mental disorders, depressive and anxiety symptoms were explored through univariable and multivariable regression analyses.

**Results:**

In total, 1203 individuals (mean age 43.7 ± 13.6 years, 48.7% male) were interviewed. Most respondents (67.8%) of the sample had already experienced Covid-19-related stressors (20.1% had Sars-CoV-2 infection; 43.2% had a close relative member who had Covid-19; 28.2% reported lack of appropriate protection; 27.5% had been quarantined) and about 50% had already been vaccinated. The prevalence of any mental disorder was 15.2% (95% CI 13.2–17.2): mood disorders 4.6%, anxiety disorders 4.3% and substance use disorders 8.0%. Mean PHQ-9 was 3.2 ± 3.8 and GAD-7 was 2.1 ± 3.1. In this study, one Covid-19 stressor, i.e. lack of protective equipment, was weakly associated with a greater frequency of anxiety disorders (*p* = 0.023), while the other stressors had significant associations with several groups of mental disorders and symptom levels.

**Conclusions:**

Our study did not provide any evidence that the prevalence of mental disorders exceeds the range of pre-pandemic data reported in the literature. Covid-related stressors, although frequently reported, did not dramatically influence the prevalence of mental disorders. The provision of the appropriate equipment at workplaces might lead to the reduction of anxiety disorders.

## Introduction

During the first year of the Covid-19 pandemic, many studies suggested an impact of the pandemic on mental health (Nochaiwong *et al*., 2021); however, assessment of the prevalence rates of mental disorders based on the in-person diagnostic interview was very rare. Maintaining physical distance during pandemics oriented the majority of researchers to conduct online surveys, which can be prone to information bias and might affect the estimates of the finding.

A longitudinal survey in the Czech Republic showed an increase in the prevalence of mental disorders from 20% before the pandemic to 30% in May 2020, and then to 33% in November 2020 (Winkler *et al*., 2020, 2021). Similarly, surveys from the United Kingdom and the United States reported increased levels of mental distress in the first months of the pandemic as compared to baseline measures before the pandemic (Daly *et al*., 2020; McGint *et al*., 2020). In the same period, representative surveys in Brazil and Norway found no increase or even a decrease in mental disorders (Brunoni *et al*., 2021; Knudsen *et al*., 2021). A meta-analysis that evaluated symptoms of depression and anxiety using PHQ-9 and GAD-7 scales showed that the global prevalence of self-rated clinically relevant levels of depression and anxiety were 24.0 and 21.3%, respectively (Castaldelli-Maia *et al*., 2021), with a wide variance reported regarding the region- and country-level.

After the prolonged pandemic, the context has subsequently changed. In the second year and beyond, populations have already experienced repeated imposition and easing of social restrictions to curb the spread of the virus, and vaccinations are available and being rolled out. Also, more people have experienced events that could be potentially stressful (being infected themselves, staying in quarantine, having a close relative who has been infected or having to work without appropriate protective equipment). In the second year, the experience of these events usually regarded as stressors was frequent and varied across the population so that their association with mental health indicators could be explored with statistical methods.

Against this background, we conducted a survey based on a representative sample of the adult population with face-to-face interviews in Serbia. Conducting a population-based study in the midst of a global pandemic was a great challenge; however, Serbia was one of the first countries with a widely rolled out vaccination programme (World Health Organization). Once the first waves of demand for vaccinations had subsided, immunisation facilities were able to handle walk-in vaccinations, allowing people from any group to come for vaccination at any time, with only an identification document required. Our data collection was done in mid-2021 at a time when strict restrictions had been imposed and lifted again, and substantial parts of the Serbian population had been vaccinated (Šiđjanin *et al*., 2021). We aimed to assess the prevalence of mental disorders and intensity of depressive and anxiety symptoms and to explore whether the experience of stressors directly related to Covid-19 was associated with the levels of disorders and symptoms. We also assessed personal characteristics (e.g., gender), and stressors not directly related to Covid-19 (e.g., relationship problems and financial difficulties) to check whether any association between Covid-19-related stressors and mental health could be explained by other factors that were not directly related to Covid-19.

## Methods

### Study design

This study (CoV2Soul.rs) was a cross-sectional observational epidemiological survey with multistage probabilistic household sampling and in-person interviews (registration number NCT04896983). Detailed information about the methodology including sampling, eligibility criteria, sample size calculation and research assistant training is reported in the protocol paper (Marić *et al*., 2021). Ethical Committees of the Faculty of Medicine (1322-VII/31), Faculty of Philosophy in Belgrade (02-33/273) and Faculty of Philosophy in Novi Sad approved the protocol (05-27, br.893/1). The study was conducted in accordance with the Declaration of Helsinki. All participants were informed of the purpose of the study and provided their informed consent.

### Setting

The study was conducted in Serbia which has a total population of 7 186 862, whose mean age is 42.2 years, and of whom 48.7% are male (Gavrilovic, 2019). Citizens have universal free access to healthcare and compensation for sick leave. Applying multistage probabilistic household sampling, respondents were recruited in 135 randomly selected local communities in 60 out of the 180 municipalities in Serbia. The data collection took place between June and October 2021, i.e. between the third and the fourth peaks of the pandemic, with limited restrictions in place at the time. According to information provided by the Ministry of Health, Republic of Serbia, at the time of recruitment, there were around 32 500 cases of confirmed Covid-19 infection in 1 034 000 tested individuals (mid-September 2021, Ministry of Health RS). The decree that prescribes mandatory measures that the employer must regulate with its plan of preventive measures, which form an integral part of the act on risk assessment, has been issued by the government (Official Gazette of RS, No 151/2020). However, when United Nations Human Rights Team in Serbia (within the UN OHCHR Surge II Initiative) explored the Impact of the Covid-19 Epidemic on the Position and Rights of Workers they noticed failures to provide recommended protective equipment in several sectors (OHCHR, 30 September 2020).

### Data collection

Data were collected by research assistants through in-person interviews. All research assistants were either psychologists, medical doctors or senior medical students and had been additionally trained in recruitment techniques, general interview skills and the application of the assessment instruments. They had also successfully completed three test assessments and were consistently supervised by senior researchers.

### Participants

Interviewees were 18–65 years of age, were residents in the identified households, spoke Serbian and provided written informed consent. In the identified local communities, households were selected in a random walk method, and the person with the most recent birthday date (which is a standard method used in studies enabling quasi-random selection of respondents (Salmon and Nichols, 1983; Lavrakas, 2008)) was asked to participate. The envisaged sample size was 1200 to detect correlations of 0.08, with a power of 0.80, at a 0.05 *α* level.

### Variables and instruments

Using a structured questionnaire, interviewees were asked about: age; gender; level of education (years of school education and in categories: elementary school, high school or vocational school, college or university); employment status (employed, unemployed, student, retired); marital status (married, single, divorced, widowed); the population size of the settlement (<20.000, 20.000–99.999, ⩾100.000); current physical disorders (cardiovascular diseases, endocrinological diseases, cancer, chronic lung disease, diabetes, chronic liver disease or kidney disease, rheumatological conditions and neurological diseases); and history of the mental disorder before the pandemic (contact with health services with a diagnosis of a mental disorder or no such contact).

We explored potential associations between mental disorders, depression and anxiety symptoms with socio-demographic characteristics, current physical illness, contact with health services because of a mental disorder before the pandemic and stressful events since the beginning of the pandemic. As Covid-19-related stressors, we considered events that could be potentially stressful as a direct result of infection of the participant or a close relative or events associated with an increased risk of Sars-CoV-2 infection. We asked the participants whether they had experienced any of the following events since the beginning of the pandemic: infection with Covid-19 (with a positive test); having a close relative with Covid-19 infection (with a positive test); obligation to stay in self-isolation for a period of time and a lack of Covid-19 protective equipment at their workplace when such equipment would have been appropriate (this could include equipment depending on the type of workplace).

We also assessed other stressors, that is, other threatening events since the beginning of the pandemic not directly related to the infection. We used a 12-item List of Threatening Events (LTE) (Brugha *et al*., 1985) and grouped items into four categories according to Motrico *et al*. (2013): illness and bereavement in close person (close friend or other relative died; serious illness, injury or assault to close relative; parent, child or spouse died); job and financial problems (major financial crisis; become unemployed/seeking work for more than one month; sacked from job); personal problems (serious illness, injury or assault to self; serious problems with close friend, neighbour or relative; something valuable lost or stolen; problems with police and court appearance); and spousal and relationship problems (broke off a steady relationship; separation due to marital problems). If participants reported that a parent, child, spouse, close friend or other relative died due to Covid-19 or that their serious illness was related to Covid-19, it was considered only as a Covid-19-related stressor (i.e., having a close relative with Covid-19 infection) and not counted in this list.

Current mental disorders and symptoms of depression and anxiety were obtained as dependent variables. Current mental disorders were observer-rated on the Mini International Neuropsychiatric Interview (MINI Standard 7.0.2.) (Sheehan *et al*., 1998) in DSM-5 diagnostic categories: major depressive episode, current manic/hypomanic episode, current psychotic episode, post-traumatic stress disorder, obsessive-compulsive disorder, social phobia, panic disorder, eating disorders, generalised anxiety disorder, agoraphobia, alcohol use disorder and substance use disorder. In addition, suicidality was assessed. The reliability of the MINI interview in the Serbian population has already been shown (Priebe *et al*., 2010). Serbian translation was provided by the official translation and linguistic validation service (Mapi Research Trust).

For the analysis as dependent variables, the diagnostic categories of current mental disorders were collapsed into three larger groups: mood disorders (major depressive episode and suicidality), anxiety disorders (panic disorder, agoraphobia, social anxiety disorder, generalised anxiety disorder, obsessive-compulsive disorder and post-traumatic stress disorder) and substance use disorder (alcohol use disorder and substance use disorder).

Symptom levels of depression and anxiety were self-rated on the Patient Health Questionnaire-9 (PHQ-9) (Kroenke *et al.*, 2001) and on General Anxiety Disorder-7 (GAD-7) (Spitzer *et al*., 2006). The PHQ-9 and GAD-7 have all been widely used in epidemiological research with well-established psychometric properties. The reliability and validity of PHQ-9 (Miletic *et al*., 2015; Subotić *et al*., 2015) and GAD-7 (Rokvić, 2019) in Serbian have been documented. We calculated a sum score, median and ranges. To interpret the findings of symptom intensity (PHQ-9 range: 0–27; GAD-7 range: 0–24) we considered the cut-off ⩾10 as clinically relevant depression or anxiety.

### Data analysis

Descriptive statistics were used to describe the sample and distribution of all variables. Univariable relations were investigated by using *χ*^2^ tests for associations between categorical variables, by ANOVAs for associations between categorical and continuous variables, and by correlations between continuous variables. Effect sizes – *φ* coefficient and Cohen's *d*, were interpreted as follows: 0.2 small, 0.5 medium, 0.8 large.

For assessing multivariable associations, the education level was turned into a continuous variable as the number of years in school education. Multivariable associations between all potential independent and dependent variables were investigated in logistic regression analyses for relations with mental disorders, and in linear regression analyses for associations with the level of depression and anxiety symptoms.

### Role of the funding source

The funders of the study had no role in the study design, data collection, data analysis, data interpretation or writing of the report.

## Results

A total of 1203 participants with a mean age of 43.7 years (s.d. = 13.6) were interviewed. To reach this number, 1796 potential participants were selected, of whom 593 could not be contacted, did not attend previously agreed interview dates or declined to be interviewed, reflecting a response rate of 67%. Missing data were rare and never exceeded 0.05% per variable. The total number of missing data for the variables analysed in this study was below 1%, commonly considered to be inconsequential (Schafer, 1999). Missing data were replaced through regression estimates. Of the interviewed participants, 48.7% were male, 59.5% were married, 57.8% were employed and with a mean duration of education 12.7 years (s.d. = 2.9). About 4.9% reported to have received a diagnosis of a mental disorder by a service in the past, and 34.2% suffered from at least one current physical illness. At least one threatening event since the beginning of the pandemic that was not directly Covid-19-related was experienced by 48.6%. In total, 67.8% reported one or more Covid-19-related stressors: 20.1% previously had a Covid-19 infection themselves, 43.2% had a close relative with an infection, 27.5% had self-isolated at least once and 28.2% reported that at least once they had to work without appropriate protective equipment. At the time of the interview, 48.8% had been vaccinated.

Details of the sample characteristics are shown in Table 2. In total, 15.2% of participants met the criteria for at least one current mental disorder (mood disorders 4.6%, anxiety disorders 4.3%, substance use disorders 8.0%). Mean levels (s.d.) of depressive (PHQ-9) and anxiety (GAD-7) symptoms were 3.2 (s.d. = 3.8) (range 0–27; median = 2.0), and 2.1 (s.d. = 3.1) (range 0–21; median 1.0), respectively. The mean scores of depressive and anxiety symptoms and prevalence of all disorders that were assessed in the interviews are shown in Table 1. Other disorders were not analysed because of the small number of cases.

**Table 1.** Prevalence of mental disorders, depressive and anxiety symptoms in the nationally representative sample of Serbian adults (*N* = 1203)

Symptoms, mean (s.d.)	
Depressive symptoms	3.2 (3.8)
Anxiety symptoms	2.1 (3.1)
Any current disorder, percentage (95% CI)	15.2 (13.2–17.2); *n* = 183
Single disorder	11.1; *n* = 134
Multiple disorders	4.1; *n* = 49
Mood disorders	
Major depressive episode	2.2 (1.40–3.08); *n* = 27
Suicidality	2.8 (1.89–3.76); *n* = 34
Anxiety disorders	
Panic disorder	0.4 (0.05–0.78); *n* = 5
Generalised anxiety disorder	1.9 (1.14–2.68); *n* = 23
Agoraphobia	0.2 (0.03–0.53); *n* = 3
Social anxiety disorder	0.5 (0.10–0.90); *n* = 6
Obsessive-compulsive disorder	1.5 (0.81–2.18); *n* = 18
Post-traumatic stress disorder	1.0 (0.43–1.56); *n* = 12
Substance use disorders	
Alcohol use disorder	7.6 (6.06–9.05); *n* = 91
Non-alcohol substance use disorder	0.9 (0.38–1.45); *n* = 11
Other	
Psychotic disorders	1.6 (0.87–2.28); *n* = 19
Manic/hypomanic episode	0.4 (0.05–-0.78); *n* = 5
Eating disorders	0.2 (0.03–0.53); *n* = 3
Any past disorder, percentage (95% CI)	14.6 (12.62–16.62); *n* = 176
Major depressive episode	11.5 (9.70–13.31); *n* = 14
Manic/hypomanic episode	1.9 (1.13–2.68); *n* = 23
Suicidality	0.7 (0.20–1.12); *n* = 8
Panic disorder	1.6 (0.87–2.28); *n* = 19
Psychotic disorders	2.4 (1.54–3.28); *n* = 29

PHQ-9, Patient Health Questionnaire; GAD-7, Generalised Anxiety Disorder; CI, confidence interval.

**Fig. 1.**
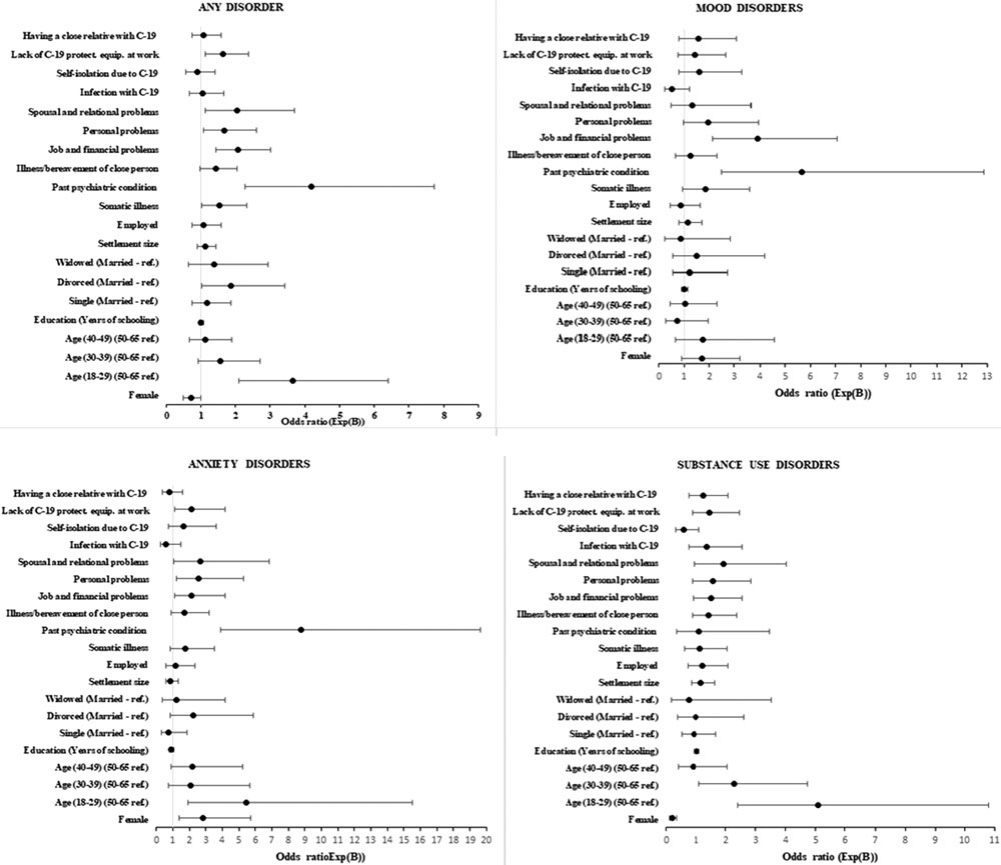
Multivariate predictions of mental disorders, odds ratios with confidence intervals.

Table 2 shows the prevalence of any mental disorder, mood disorders, anxiety disorders, substance use disorders and the levels of depression and anxiety symptoms dependent on the categories of all the considered independent variables. The table also indicates which univariable associations are statistically significant ( ). Table 3 and Fig. 1 show the findings of the multivariable associations.

**Table 2.** Prevalence of current mental disorders and level of symptoms by sociodemographic characteristics, health, stressors not directly related to Covid-19 and Covid-19-related stressors

Total (*N* = 1203)	Any disorder (*N* = 183)	Mood disorders (*N* = 55)	Anxiety disorders (*N* = 52)	SUD (*N* = 96)	PHQ-9 (*N* = 1203)	GAD-7 (*N* = 1203)
Gender, *n* (%)	*n* (%)				Mean (s.d.)	
Male, 586 (48.7)	101 (17.2)	19 (3.2)	14 (2.4)	79 (13.5)	2.6 (3.4)	1.6 (2.7)
Female, 617 (51.3)	82 (13.3)	36 (5.8)	38 (6.2)	17 (2.8)	3.8 (4.0)	2.6 (3.4)
Gender differences*	*p* = 0.057	***p* = 0**.**031**	***p* = 0**.**002**	***p* < 0**.**001**	***p* < 0**.**001**	***p* < 0**.**001**
Age categories, *n* (%)	*n* (%)				Mean (s.d.)	
18–29, 232 (19.3)	60 (25.9)	14 (6.0)	14 (6.0)	40 (17.2)	3.4 (3.4)	2.5 (2.9)
30–39, 246 (20.4)	36 (14.6)	7 (2.8)	9 (3.7)	25 (10.2)	3.0 (3.5)	2.0 (3.0)
40–49, 261 (21.8)	31 (11.9)	13 (5.0)	14 (5.4)	10 (3.8)	3.0 (4.2)	1.9 (3.4)
50–65, 464 (38.5)	56 (12.1)	21 (4.5)	15 (3.2)	21 (4.5)	3.3 (3.8)	2.0 (3.1)
Differences between the age groups*	***p* < 0**.**001**	*p* = 0.404	*p* = 0.270	***p* < 0**.**00**	*p* = 0.179	*p* = 0.388
Education categories, *n* (%)	*n* (%)				Mean (s.d.)	
Elementary school, 302 (25.1)	47 (15.6)	18 (6.0)	18 (6.0)	19 (6.3)	3.4 (4.3)	2.4 (3.6)
High school, and vocational school, 639 (53.1)	95 (14.9)	23 (3.6)	27 (4.2)	54 (8.5)	3.2 (3.6)	2.0 (2.9)
College or university, 262 (21.8)	41 (15.6)	14 (5.3)	7 (2.7)	23 (8.8)	3.1 (3.5)	2.0 (2.9)
Differences between the education categories*	*p* = 0.939	*p* = 0.215	*p* = 0.157	*p* = 0.451	*p* = 0.584	*p* = 0.196
Marital status, *n* (%)	*n* (%)				Mean (s.d.)	
Married, 716 (59.5)	81 (11.3)	28 (3.9)	25 (3.5)	42 (5.9)	3.1 (3.6)	1.9 (2.9)
Single, 329 (27.3)	70 (21.3)	17 (5.2)	13 (4.0)	46 (14.0)	3.2 (3.6)	2.2 (3.0)
Divorced, 89 (7.4)	21 (23.6)	6 (6.7)	9 (10.1)	6 (6.7)	3.4 (4.4)	2.9 (4.1)
Widowed, 69 (5.8)	11 (15.9)	4 (5.8)	5 (7.2)	2 (2.9)	4.3 (5.0)	2.5 (3.9)
Differences between the categories*	***p* < 0**.**001**	*p* = 0.533	***p* = 0**.**019**	***p* < 0**.**001**	*p* = 0.087	***p* = 0**.**046**
Employment status, *n* (%)	*n* (%)				Mean (s.d.)	
Employed, 695 (57.8)	95 (13.7)	23 (3.3)	24 (3.5)	58 (8.3)	3.0 (3.6)	1.9 (2.9)
Other, 508 (42.2)	88 (17.3)	32 (6.3)	28 (5.5)	38 (7.5)	3.6 (4.0)	2.4 (3.3)
Differences between the employment status*	*p* = 0.081	***p* = 0**.**014**	*p* = 0.083	*p* = 0.584	***p* = 0**.**008**	***p* = 0**.**006**
Size of settlement, *n* (%)	*n* (%)				Mean (s.d.)	
Small (up to 19 999 inhabitants), 352 (29.4)	51 (14.5)	12 (3.4)	15 (4.3)	28 (8.0)	2.8 (3.5)	1.9 (2.9)
Medium (20 000–99 999 inhabitants), 564 (46.8)	76 (13.5)	26 (4.6)	24 (4.3)	37 (6.6)	3.2 (3.8)	2.0 (3.1)
Large (1 00 000+ inhabitants), 287 (23.8)	56 (19.5)	17 (5.9)	13 (4.5)	31 (10.8)	3.7 (3.9)	2.6 (3.3)
The comparison between the settlements*	*p* = 0.062	*p* = 0.318	*p* = 0.981	*p* = 0.097	***p* = 0**.**006**	***p* = 0**.**005**
Other variables						
Health, *n* (%)	*n* (%)*				Mean (s.d.)*	
Psychiatric diagnosis (past), 59 (4.9)	**22 (37.3) *p* < 0**.**001**	**11 (18.6) *p* < 0**.**001**	**14 (23.7) *p* < 0**.**001**	4 (6.8) *p* = 0.727	**6.8 (7.1) *p* < 0**.**001**	**5.1 (5.9) *p* < 0**.**001**
Current somatic illness (yes), 412 (34.2)	65 (15.8) *p* = 0.694	**26 (6.3) *p* = 0**.**037**	23 (5.6) *p* = 0.121	**22 (5.3) *p* = 0**.**015**	**3.8 (4.0) *p* < 0**.**001**	2.3 (3.4) *p* = 0.121
Vaccination status (vaccinated), 587 (48.8)	92 (15.7) *p* = 0.664	29 (4.9) *p* = 0.550	26 (4.4) *p* = 0.859	46 (7.8) *p* = 0.858	3.3 (3.8) *p* = 0.291	2.1 (3.1) *p* = 0.955
Stressors not directly related to Covid-19, *n* (%)	*n* (%)*				Mean (s.d.)*	
Illness and bereavement of close person (yes), 320 (26.6)	**65 (20.3) *p* < 0**.**003**	**22 (6.9) *p* = 0.021**	**23 (7.2) *p* < 0**.**004**	31 (9.7) *p* < 0.188	**3.9 (4.0) *p* < 0**.**001**	**2.8 (3.7) *p* < 0**.**001**
Job and financial problems (yes), 260 (21.6)	**66 (25.4) *p* < 0**.**001**	**29 (11.2) *p* < 0**.**002**	**22 (8.5) *p* < 0**.**001**	**29 (11.1) *p* = 0**.**034**	**4.5 (5.2) *p* = 0**.**003**	**3.4 (3.6) *p* = 0**.**001**
Personal problems (yes), 154 (12.8)	**41 (26.6) *p* < 0**.**001**	**15 (9.7) *p* < 0**.**001**	**16 (10.4) *p* < 0**.**001**	**21 (13.6) *p* = 0**.**006**	**5.0 (4.9) *p* < 0**.**001**	**3.2 (4.1) *p* < 0**.**001**
Spousal and relational problems (yes), 66 (5.5)	**24 (36.4) *p* = 0**.**003**	6 (9.1) *p* = 0.071	**9 (13.6) *p* = 0**.**001**	**14 (21.2) *p* = 0.001**	**3.5 (3.8) *p* = 0.008**	**2.3 (3.2) *p* = 0**.**036**
Covid-19-related stressors, *n* (%)	*n* (%)*				Mean (s.d.)*	
Infection with Covid-19, 242 (20.1)	35 (14.5) *p* = 0.717	9 (3.7) *p* = 0.477	8 (3.3) *p* = 0.384	20 (8.3) *p* = 0.855	3.3 (3.6) *p* = 0.862	2.1 (3.0) *p* = 0.965
Self-isolation for a period of time, 331 (27.5)	49 (14.8) *p* = 0.808	21 (6.3) *p* = 0.070	18 (5.4) *p* = 0.241	21 (6.3) *p* = 0.197	3.5 (4.0) *p* = 0.101	2.2 (3.3) *p* = 0.506
Lack of protective equipment at workplace, 339 (28.2)	**64 (18**.**9) *p* = 0**.**027**	19 (5.6) *p* = 0.283	**21 (6**.**2) *p* = 0**.**045**	31 (9.1) *p* = 0.351	3.3 (4.0) *p* = 0.642	2.3 (3.3) *p* = 0.121
Having a close relative with Covid-19 infection, 520 (43.2)	82 (15.8) *p* = 0.639	30 (5.8) *p* = 0.083	21 (4.0) *p* = 0.672	46 (8.8) *p* = 0.333	**3.5 (3.8) *p* = 0**.**008**	**2.3 (3.2) *p* = 0**.**036**

PHQ-9, Patient Health Questionnaire; GAD-7, Generalised Anxiety Disorder.

*p values – for any disorder, mood disorder, anxiety disorder and SUD comparison with ‘no disorder’ group; for PHQ-9 and GAD-7 comparisons on the whole sample with respect to specific variables.

Bold text indicates a *p*-value less than 0.05.

**Table 3.** Multivariable relationships between current disorders and symptoms with sociodemographic characteristics, health, stressors not directly related to Covid-19 and Covid-19-related stressors

	Any disorder (*N* = 183)		Mood disorder (*N* = 55)		Anxiety disorder (*N* = 52)		Substance use disorders (*N* = 96)		PHQ-9 (*N* = 1203)		GAD-7 (*N* = 1203)	
	Nagelkerke *R*^2^ = 15.6		Nagelkerke *R*^2^ = 18.2		Nagelkerke *R*^2^ = 24.4		Nagelkerke *R*^2^ = 20.2		*R*^2^ = 14.6		*R*^2^ = 15.8	
	B coeff.	*p* value (Wald)	B coeff.	*p* value (Wald)	B coeff.	*p* value (Wald)	B coeff.	*p* value (Wald)	*β* coeff.	*p* value	*β* coeff.	*p* value
Gender (male – ref)												
Female	−0.34	0.058 (3.6)	0.55	0.087 (2.9)	1.03	**0.004 (8.1)**	−1.67	**0.001 (32.8)**	0.15	**0.001**	0.16	**0**.**001**
Age (50–65 – ref)												
18–29	1.30	**0.001 (20.7)**	0.56	0.258 (1.3)	1.70	**0.001 (10.2)**	1.62	**0.001 (17.8)**	0.04	0.252	0.09	**0.01**
30–39	0.45	0.103 (2.7)	−0.32	0.532 (0.4)	0.72	0.161 (2.0)	0.83	**0.024 (5.1)**	0.00	0.907	0.03	0.444
40–49	0.12	0.646 (0.2)	0.04	0.928 (0.0)	0.77	0.086 (3.0)	−0.10	0.812 (0.1)	−0.10	0.683	0.00	0.962
Duration of education	0.01	0.864 (0.0)	0.03	0.614 (0.3)	−0.08	0.178 (1.8)	0.01	0.826 (0.0)	−0.30	0.227	−0.05	0.093
Marital status (married – ref)												
Single	0.17	0.470 (0.5)	0.21	0.615 (0.3)	−0.27	0.549 (0.4)	−0.06	0.844 (0.0)	0.01	0.678	0.01	0.785
Divorced	0.63	**0.040 (4.2)**	0.42	0.420 (0.6)	0.81	0.102 (2.7)	0.00	0.993 (0.0)	−0.02	0.536	0.04	0.112
Widowed	0.32	0.401 (0.7)	−0.15	0.801 (0.1)	0.22	0.726 (0.1)	−0.26	0.739 (0.1)	0.02	0.386	0.00	0.971
Employment status (employed – ref)												
Other	0.08	0.681 (0.2)	−0.15	0.647 (0.2)	0.14	0.691 (0.2)	0.21	0.439 (0.6)	0.02	0.472	0.02	0.469
Settlement size (1 – small; 2 – medium; 3 – large)	0.13	0.282 (1.2)	0.15	0.442 (0.6)	−0.14	0.524 (0.4)	0.16	0.319 (1.0)	0.06	**0.035**	0.05	0.060
Health												
Psychiatric diagnosis (past)	1.43	**0.001 (21.2)**	1.73	**0.001 (17.1)**	2.17	**0.001 (27.9)**	0.11	0.843 (0.0)	0.19	**0.001**	0.19	**0.001**
Current somatic illness	0.43	**0.038 (4.3)**	0.62	0.072 (3.2)	0.55	0.126 (2.3)	0.12	0.685 (0.2)	0.08	**0.011**	0.02	0.409
Stressors not directly related to Covid-19												
Illness and bereavement of close person	0.35	0.060 (3.5)	0.23	0.470 (0.5)	0.52	0.110 (2.6)	0.36	0.165 (1.9)	0.04	0.113	0.07	**0.008**
Job and financial problems	0.73	**0.001 (14.8)**	1.36	**0.001 (19.7)**	0.77	**0.022 (5.3)**	0.42	0.110 (2.5)	0.17	**0.001**	0.19	**0.001**
Personal problems	0.51	**0.024 (5.1)**	0.67	0.060 (3.5)	0.94	**0.011 (6.5)**	0.46	0.124 (2.4)	0.14	**0.001**	0.09	**0.001**
Spousal and relational problems	0.72	**0.017 (5.7)**	0.29	0.575 (0.3)	0.98	**0.039 (4.2)**	0.66	0.080 (3.1)	0.05	0.078	0.05	0.087
Covid-19-related stressors												
Infection with Covid-19	0.05	0.843 (0.0)	−0.64	0.137 (2.2)	−0.51	0.271 (1.2)	0.32	0.294 (1.1)	−0.02	0.538	0.00	0.971
Self-isolation for a period of time	−0.11	0.639 (0.2)	0.48	0.182 (1.8)	0.51	0.208 (1.6)	−0.52	0.099 (2.7)	0.02	0.593	−0.01	0.822
Lack of protective equipment at workplace	0.50	**0.009 (6.8)**	0.35	0.272 (1.2)	0.76	**0.023 (5.2)**	0.39	0.137 (2.2)	0.03	0.249	0.06	**0.024**
Having a close relative with Covid-19 infection	0.08	0.690 (0.2)	0.46	0.170 (1.9)	−0.25	0.501 (0.5)	0.24	0.348 (0.9)	0.06	0.054	0.05	0.084

PHQ-9, Patient Health Questionnaire; GAD-7, Generalised Anxiety Disorder; *R*^2^, multiple correlation coefficient – squared.

Bold text indicates a *p*-value less than 0.05.

In univariable analyses, the experience of two Covid-19-related stressors was linked to poorer mental health. Interviewees who have had to work without appropriate protective equipment had a higher prevalence of anxiety disorders (*p* = 0.045; *φ* coefficient = 0.06) and any mental disorder (*p* = 0.027; *φ* coefficient = 0.06), and participants with a family member who had a Covid-19 infection had higher levels of depression and anxiety symptoms (*p* = 0.008 and 0.036; Cohen's *d* = 0.16 and 0.12, respectively).

However, when the influence of all potential Covid-19-related stressors was considered in multivariable analyses, lack of protective equipment at work was the only event that was significantly associated with any of the dependent variables. Participants with such experience had higher rates of any disorder (*p* = 0.009), anxiety disorders (*p* = 0.023) and increased anxiety symptoms (*p* = 0.024).

In addition to that, several other variables showed significant associations with one or more mental health indicators in multivariable regressions. Women had a higher prevalence of anxiety disorders and more symptoms of depression and anxiety, but a lower prevalence of substance use disorders. People aged 18–29 years had higher rates of any disorder, anxiety disorders and substance use disorders, as well as more anxiety symptoms, whilst the second-youngest group with an age of 30–39 years had a higher prevalence of substance use disorders. Divorced participants had a higher prevalence of any disorder; interviewees living in larger settlements had more symptoms of depression. Those living in larger settlements and those with a current physical illness had increased symptoms of depression. Participants with the current physical illness had higher rates of any disorder.

The two variables with the strongest consistent influence were the experience of stressors since the beginning of the pandemic that was not directly related to Covid-19 and a history of a diagnosis of a mental disorder. A past diagnosis of a mental disorder was associated with poorer mental health on all indicators other than substance use disorders. Each of the four groups of stressors that were not directly linked to Covid-19 was associated with the prevalence of some disorders or symptom levels or both. In all cases, the experience of more stressors was linked to poorer mental health.

## Discussion

This was the first nationally representative study of mental disorders, depressive and anxiety symptoms and the experience of Covid-19-related stressors during the second year of the pandemic. Our study did not provide evidence that the prevalence of mental disorders exceeds the range of pre-pandemic data reported in the literature, nor that the levels of depressive and anxiety symptoms reach clinically relevant intensity. Only lack of protective equipment was associated with anxiety disorders and this association was weakly significant; however, it was held true when the influence of other variables was adjusted for in multivariable analyses. There was no evidence that other events that could be potentially stressful, such as the personal experience of infection or having a relative with Covid-19, were linked with poorer mental health once the influence of other variables was also considered. However, other stressors not directly related to the risk of the infection had significant associations with several groups of mental disorders and with the symptom levels.

The survey used a rigorous method for sampling participants, in-person interviews conducted by trained researchers and standardised instruments. It considered many potential associates in multivariable analyses and had a very low percentage of missing data but it also has several limitations. First, given the sampling method, the results apply only to people who have a fixed residency and speak the national language. The excluded populations – homeless people, transient migrants and those without sufficient command of Serbian – might be more vulnerable to developing mental disorders in response to Covid-19-related stressors. Second, conducting a face-to-face study in the midst of the pandemic presented many challenges. About 33% of the intended interviewees were not available despite repeated attempts to arrange an interview or declined to be interviewed and the given response rate could affect the generalisability of our findings. However, it has been suggested that a potential bias because of varying response rates in population surveys might not substantially alter the established prevalence rates (Morton *et al*., 2012; Kawakami *et al*., 2020). Moreover, our main research question was about the associations, and such associations are considered more robust against selection bias than prevalence rates (Etter and Perneger, 2000). Third, information about all considered stressors and about having been diagnosed with a mental disorder in services prior to the pandemic was obtained from self-reports only, which means they could have been influenced by memory or reporting bias. Like in any other study using self-report measures, we are assessing the perception a person has on a topic of interest. Finally, because of the exploratory nature of the study, a number of tests were conducted and were not adjusted for multiple testing.

The prevalence of mental disorders and levels of depression and anxiety symptoms were established in a cross-sectional survey and cannot be directly compared with similar findings in Serbia from before the pandemic. However, the overall prevalence rate of any mental disorder 15.2% is slightly lower than the global 12-month prevalence of common mental disorders (Steel *et al*., 2014), and within the range of 10–19% reported by the World Mental Health Survey Initiative (Kessler *et al*., 2009). The established prevalence of mental disorders contrasts with much higher rates found in a 2005/6 study in Serbia focusing on people with potentially stressful experiences during the previous war in the Balkans (Priebe *et al*., 2010). That survey used a similar sampling procedure to ours, and also assessed disorders through in-person interviews on the MINI. The prevalence of any mental disorder was 54.0%, mood disorders 35.9%, anxiety disorders 39.7% and substance use disorders 9.0%. This shows that the methods for assessing mental disorders applied in our study are sensitive to capturing large differences in prevalence rates in different historical contexts in Serbia. The prevalence of mental disorders in Serbia during 2021 was very similar to those in Norway during 2019–20 (Knudsen *et al*., 2021), but substantially lower than those reported in the Czech Republic during 2020 (Winkler *et al*., 2020). The mean levels of the PHQ-9 in our study are similar to the pre-pandemic results collected internationally (Kocalevent *et al*., 2013) and locally (Subotić *et al*., 2015).

One can only speculate as to whether the prevalence of disorders and levels of depressive and anxiety symptoms in Serbia were higher in the first year of the Covid-19 pandemic and then dropped or whether they had consistently been low. However, evidence from longitudinal studies suggests that, after a disaster, mental health symptoms tend to peak in the year following the disaster and then improve (Goldmann and Galea, 2014). Various factors might explain a possible improvement of mental health during the pandemic over time. More than a year after the beginning of the pandemic, people may have adjusted their everyday lives to varying restrictions and the ongoing threat to their own health and the health of others around them. Vaccination programmes might have instilled hope that the pandemic can be overcome, and individuals might have built up their resilience by discovering new resources and personal strengths.

The low prevalence of mental disorders and the low level of depression and anxiety symptoms make it unlikely that the pandemic has had a strong overall negative influence on mental health. Except for lack of the protective equipment, all other events that we considered as Covid-19 stressors were experienced more frequently than other threatening events but did not show significant associations with mental disorders, or – when the influence of other factors was considered in multivariable analyses – with either level of depression or anxiety.

Other threatening events since the beginning of the pandemic had strong associations with mental disorders and symptom levels. These stressful experiences, such as personal problems, job related and financial difficulties, are known to have a potential negative influence on mental health in any context and at any time (Lund *et al*., 2018). The processes behind these stressors are often complex, and some of them may have been indirectly influenced by the consequences of and societal responses to the pandemic. However, it would be difficult in individual cases to establish whether and, if so, to what extent these stressors were directly caused or influenced by the pandemic. In contrast, all stressors that we considered as Covid-19-related in this study were clearly and directly linked to an increased risk of Sars-CoV-2 infection.

At the time of the assessment, almost half of the study sample was vaccinated, more than a year passed after the first lockdown and on-and-off restrictions were milder as compared to the initial outbreak. It is therefore likely that future stages of the pandemic will resemble the context of this survey to some extent.

The findings have potential implications for research, clinical practice and policies. The pandemic is ongoing. As compared to studies conducted in the first year of the pandemic, the context of this study is likely to resemble more current and future societal conditions. The findings may be a reason for cautious optimism that Covid-19-related stressors will not lead to substantial deterioration of mental health across the adult population. Clinicians may want to consider that there is no evidence suggesting that infections of patients themselves or of their relatives lead to poorer mental health. However, they should be aware that the experience of working without appropriate personal protective equipment for Covid-19 might be associated with anxiety disorders.

Future surveys on the impact of Covid-19-related stressors should preferably be representative and adjust for the influence of other stressors not directly related to Covid-19 to avoid misleading positive associations between Covid-19-related stressors and mental conditions.

## Data Availability

The dataset supporting our findings is publicly shared on OSF: https://osf.io/f8sje/
